# Maternal Obesity Affects the Glucose-Insulin Axis During the First Trimester of Human Pregnancy

**DOI:** 10.3389/fendo.2020.566673

**Published:** 2020-10-09

**Authors:** Julia Bandres-Meriz, Anna M. Dieberger, Denise Hoch, Caroline Pöchlauer, Martina Bachbauer, Andreas Glasner, Tobias Niedrist, Mireille N. M. van Poppel, Gernot Desoye

**Affiliations:** ^1^Department of Obstetrics and Gynecology, Medical University of Graz, Graz, Austria; ^2^Femina-Med Center, Graz, Austria; ^3^Clinical Institute of Medical and Chemical Laboratory Diagnostics, Medical University of Graz, Graz, Austria; ^4^Institute of Human Movement Science, Sport and Health, University of Graz, Graz, Austria

**Keywords:** first trimester pregnancy, obesity, fat mass, glucose, C-peptide, insulin sensitivity

## Abstract

**Background and objective:** The maternal glucose-insulin axis is central for metabolic adaptations required for a healthy pregnancy. Metabolic changes in obese mothers in early pregnancy have been scantly described. Here we characterized the glucose-insulin axis in the first trimester of human pregnancy and assessed the effect of maternal obesity and fat mass.

**Methods:** In this cross-sectional study, maternal blood samples (*N* = 323) were collected during voluntary pregnancy termination (gestational age 4^+0^–11^+6^ weeks) after overnight fasting. Smokers (*N* = 198) were identified by self-report and serum cotinine levels (ELISA). Maternal BMI (kg/m^2^) and serum leptin (ELISA) were used as proxy measures of obesity and maternal fat mass, respectively. BMI was categorized into under-/normal weight (BMI < 25.0 kg/m^2^), overweight (BMI 25.0–29.9 kg/m^2^) and obese (BMI ≥ 30.0 kg/m^2^), and leptin in tertiles (1st tertile: leptin < 6.80 ng/ml, 2nd tertile: leptin 6.80–12.89 ng/ml, 3rd tertile: leptin > 12.89 ng/ml). IS_HOMA_ insulin sensitivity index was calculated from glucose and C-peptide (ELISA) serum concentrations. Analyses of covariance including multiple confounders were performed to test for differences in glucose, C-peptide and IS_HOMA_ between gestational age periods, BMI and leptin groups. C-peptide and IS_HOMA_ were log-transformed before analyses.

**Results:** At weeks 7–9, fasting glucose and C-peptide levels were lower (*P* < 0.01 and *P* < 0.001, respectively) and insulin sensitivity higher (*P* < 0.001) than at weeks 4–6. Glucose levels were not significantly different between BMI or leptin categories. In contrast, C-peptide increased by 19% (*P* < 0.01) between the normal weight and the overweight group and by 39% (*P* < 0.001) between the overweight and obese group. In the leptin groups, C-peptide increased by 25% (*P* < 0.001) between the 1st and 2nd leptin tertile and by 15% (*P* < 0.05) between the 2nd and 3rd leptin tertile. IS_HOMA_ decreased with higher BMI and fat mass. IS_HOMA_ decreased by 18% (*P* < 0.01) between the normal weight and the overweight group and by 30% (*P* < 0.01) between the overweight and the obese group. In the leptin groups, IS_HOMA_ decreased by 22% (*P* < 0.001) between the 1st and 2nd leptin tertile and by 14% (*P* < 0.05) between the 2nd and 3rd leptin tertile.

**Conclusions:** At the group level, fasting glucose, C-peptide and insulin sensitivity dynamically change in the first trimester of human pregnancy. Maternal obesity is associated with higher C-peptide and lower insulin sensitivity at all periods in the first trimester of human pregnancy, while glucose is unaltered. These findings have implications for the timing of early gestational diabetes mellitus risk screening.

## Introduction

The first trimester of pregnancy is a critical period for placentation and early cell differentiation, making it especially sensitive to environmental changes (1). The timing of the exposure to adverse metabolic influences is crucial since it will affect organogenesis (2). Therefore, any dysregulation in the maternal metabolism already in the first trimester of pregnancy may result in pregnancy complications (3, 4). Maternal metabolic disturbances such as obesity and type 2 diabetes mellitus (T2DM) are associated with decreased fetal growth early in pregnancy, followed by a catch-up growth (5, 6), suggesting a central role of the maternal glucose-insulin axis in the metabolic and endocrine adaptations required for a healthy pregnancy. Indeed, impaired fasting glucose levels at weeks 9–10 positively associate with an increased risk of developing gestational diabetes mellitus (GDM) and giving birth to large for gestational age (LGA) offspring (7). Obesity is well-known to modify the glucose-insulin axis, as it often goes hand in hand with hyperinsulinemia and insulin resistance (8). This poses a risk for adverse pregnancy outcomes. However, despite their importance, metabolic changes in obese mothers early in pregnancy have been poorly described, either in small cohorts, at only a short time period within the first trimester or not fully addressing the glucose-insulin axis (9, 10). Indirect evidence in women with T1DM, whose insulin requirement declines at the end of the first trimester of pregnancy, suggests a change in insulin sensitivity in this early pregnancy period (11, 12). To the best of our knowledge, no one has systematically studied changes in insulin as well as insulin sensitivity during the first trimester, and potential influences of maternal obesity on those changes. We hypothesized that the glucose-insulin axis in the first trimester of human pregnancy differs depending on maternal obesity status. Hence, in the present cross-sectional study we aimed to analyse the influence of maternal obesity on the glucose-insulin axis in the first trimester of human pregnancy spanning the range of week 4^+0^ to 11^+6^. Because of its well-known effects on insulin sensitivity (13, 14), we objectively ascertained maternal smoking and included it as an important potential confounder.

## Materials and Methods

### Study Population and Design

The study was approved by the ethical committee of the Medical University of Graz (no.31-094 ex 18/19). All participants provided written informed consent after full explanation of the purpose and nature of all procedures.

This prospective, cross-sectional study was conducted in a non-academic setting between May 2017 and August 2018. It included 323 pregnant women, who underwent voluntary pregnancy termination (gestational age 4^+0^–11^+6^ weeks). Pregnant women ≥ 18 years old with a singleton pregnancy were included, women with known co-morbidities, e.g., pre-existing diabetes mellitus, were excluded. Information on age and smoking were self-reported by the participants. Height (centimeters) and weight (kilograms) were measured before pregnancy termination and used to calculate body mass index (BMI; kg/m^2^) as an indicator of maternal obesity. Gestational age was defined as days post last menstrual period (LMP) and corroborated by ultrasound measurement of crown-rump length (CRL). Self-reported non-smoking status was complemented by quantification of serum cotinine levels. If cotinine levels were above the threshold (cut-off of ≤ 0.03 nmol/l) (15), women were classified as smokers independently of the self-reported status. Cohort characteristics are shown in Table 1.

**Table 1:** Characteristics of the women participating in the study stratified by BMI.

**Maternal characteristics**	**All**	**Stratified by BMI**			***P***
	***N* = 323**	**Under-/normal weight *N* = 227**	**Overweight *N* = 72**	**Obese *N* = 24**	
Age, *years* (mean ± SD, *N* = 322)	29.5 ± 7.0	29.3 ± 7.0	30.4 ± 7.2	28.6 ± 5.9	0.413
Smokers [*N* (%)]	198 (61.3)	143 (63.0)	39 (54.2)	16 (66.7)	0.348
Gestational age [*N* (%)]					**0.018**
4–6 weeks	134 (41.5)	90 (39.6)	32 (44.4)	12 (50.0)	
7–9 weeks	138 (42.7)	108 (47.6)	21 (29.2)	9 (37.5)	
10–12 weeks	51 (15.8)	29 (12.8)	19 (26.4)	3 (12.5)	
BMI, *kg/m^*2*^* [median (IQR)]	22.7 (20.5–25.4)	21.3 (19.7–22.9)	26.8 (25.7–28.3)	32.3 (31.1–35.3)	** <0.001**
Leptin, *ng/ml* [median (IQR), *N* = 321]	9.6 (5.0–15.8)	8.0 (4.0–11.7)	16.2 (9.9–18.6)	20.6 (16.4–29.1)	** <0.001**
Fasting glucose, *mmol/l* (mean ± SD)	4.7 ± 0.8	4.7 ± 0.8	4.8 ± 0.8	5.0 ± 0.9	0.129
Fasting C-peptide, *pmol/l* [median (IQR), *N* = 322]	357.7 (270.1–454.8)	329.1 (258.0–422.1)	414.4 (301.6–404.3)	545.9 (431.7–680.8)	** <0.001**
IS_HOMA_ [median (IQR), *N* = 322]	0.76 (0.55–1.04)	0.82 (0.61–1.08)	0.68 (0.49–0.89)	0.50 (0.38–0.68)	** <0.001**
IS_20/(FCPxFPG)_ [median (IQR), *N* = 322]	12.10 (8.85–16.62)	13.12 (9.79–17.35)	10.94 (7.92–14.31)	8.02 (6.04–10.81)	** <0.001**
IS_QUICKI_ (mean ± SD, *N* = 322)	0.22 ± 0.01	0.23 ± 0.01	0.22 ± 0.01	0.21 ± 0.21	** <0.001**

*BMI, Body mass index (BMI < 25.0 kg/m^2^: under-/normal weight; BMI 25.0–29.9 kg/m^2^: overweight; BMI ≥ 30.0 kg/m^2^: obese); Gestational age, postmenstrual period; IS_HOMA_, Homeostatic model assessment of insulin sensitivity; IQR, Interquartile range; SD, Standard deviation. Skewed variables were log-transformed before analysis. Statistically significant results in bold*.

### Blood Collection and Storage

Venous blood (8 ml) was collected after overnight fasting in S-Monovette® (Sarstedt, Nümbrecht, Germany REF.: 02.1063, clot activator) collection tubes and centrifuged at 2,000 × g at 4°C for 10 min after arrival in the laboratory. After centrifugation, the serum fraction was aliquoted and immediately frozen at −80°C. Processing time, defined as the time (minutes) between blood collection and centrifugation of the sample in the laboratory, was carefully recorded.

### Cotinine, Leptin, C-Peptide and Glucose Assays

Serum cotinine levels were measured with a competitive immunoassay (Abnova, Taipei, Taiwan Cat# KA0930) using a cut-off of ≤ 0.03 nmol/l (15) cotinine for smokers. Analytical sensitivity of the assay was 1 ng/ml, cross-reactivities: nicotine <1%, nicotinamide <1%, nicotinic acid <1%.

Leptin (ng/ml), used as a proxy for maternal fat mass, was measured by a sandwich immunoassay (DRG, Marburg, Germany, Cat# EIA2395). Intra-assay and inter-assay CVs were 6.2 and 6.6%, respectively. The analytical sensitivity was 0.7 ng/ml and recovery was 93.5% with no cross-reactivity with human insulin, proinsulin, C-peptide, glucagon or IGF-I. Serum C-peptide (pmol/l) was measured by a Sandwich Immunoassay (R&D Systems Minneapolis, USA Cat# DICP00). Intra-assay and inter-assay CVs were 3.1 and 8.3%, respectively. The analytical sensitivity was 2.88 pmol/l and recovery was 100.4%. Cross-reactivity of <0.5% was observed with recombinant human IGF I, IGF II, insulin, proinsulin, and relaxin.

Serum glucose (mmol/l) was measured using hexokinase-based test (Glucose HK Gen.3, Roche Diagnostics, Mannheim, Germany) on an automated analyzer (cobas® 8000 c701, Roche Diagnostics, Mannheim, Germany). Previous studies showed a 7% per hour decrease in glucose levels in whole blood (16) due to glucose consumption by erythrocytes. Therefore, glucose values were corrected accordingly for processing time.

### Outcomes

Main outcomes were fasting serum glucose, fasting C-peptide and insulin sensitivity. Fasting serum glucose and fasting C-peptide concentrations were used to calculate insulin sensitivity based on three indices: Homeostatic model assessment of insulin sensitivity (IS_HOMA_), IS_QUICKI_ and IS_20/(FCPxFPG)_ (17, 18), using the following formulas:

$$\begin{array}{l} ISHOMA=\;\frac{22.5}{Cpeptide\left(pmol/l\right)\times glucose\;\left(mg/dl\right)}\\ ISQUICKI=\frac{1}{\log Cpeptide\left(pmol/l\right)+\log glucose\;\left(mg/dl\right)}\\ IS\frac{20}{FCP\times FPG}=\frac{20}{Cpeptide\;\left(nmol/l\right)\times glucose\;\left(mmol/l\right)} \end{array}$$

### Statistical Analyses

Normal distribution of data was assessed visually with histograms and QQ-plots and by comparison of mean and median values. Skewed data were log-transformed before being used in statistical models and re-transformed for the presentation of results.

For description of baseline characteristics, mean and standard deviation (SD) were calculated for normally distributed continuous variables, median and interquartile range (IQR) were calculated for continuous variables without normal distribution. Categorical variables are described by frequencies and percentages. BMI and leptin are presented as both continuous and categorical variables. BMI was categorized into under- and normal weight (BMI < 25.0 kg/m^2^) overweight (25.0–29.9 kg/m^2^) and obesity (≥ 30.0 kg/m^2^) (19). In absence of established cut-off points for leptin and to match with the three BMI categories, leptin levels were categorized in tertiles (1st tertile leptin < 6.80 ng/ml, 2nd tertile leptin 6.80–12.89 ng/ml and 3rd tertile leptin > 12.89 ng/ml). Baseline characteristics are presented for all participants combined as well as stratified by BMI group and smoking status. Differences between the groups were tested by analysis of variance (ANOVA) for continuous variables and chi-square test for categorical variables.

To assess whether the outcome variables glucose, C-peptide and insulin sensitivity are associated linearly with gestational age, leptin and BMI, the outcome variables were stratified by gestational age (weeks 4^+0^–6^+6^, weeks 7^+0^–9^+6^, and weeks 10^+0^–11^6^), the BMI groups and leptin tertiles. As changes in the outcome variables between the gestational age groups, BMI and leptin groups were non-linear, all variables were kept categorical for all further analyses.

ANOVA was used to assess differences of fasting glucose, fasting C-peptide and IS_HOMA_, between gestational age periods (4–6 weeks, 7–9 weeks, 10–12 weeks). To examine whether the associations are influenced by covariates, analyses were repeated as analysis of covariance (ANCOVA), adjusting for BMI (categorical), maternal age (years; continuous), smoking (yes/no; dichotomous), and processing time (minutes; continuous).

Associations between maternal obesity (BMI) or fat mass (leptin) and metabolic parameters (fasting glucose, fasting C-peptide and IS_HOMA_) were examined using ANCOVA, including the a-priori defined covariates gestational age (4–6 weeks, 7–9 weeks, 10–12 weeks; categorical), maternal age (years; continuous), smoking (yes/no; dichotomous), and processing time (minutes; continuous). Equal variances of variables were verified by Levene's test. Results are presented as estimated marginal means and 95% confidence intervals.

Interactions between gestational age and smoking, gestational age and leptin and gestational age and BMI were tested, respectively, by including both variables and all covariates in the ANCOVA model and adding an interaction term. An interaction term was deemed significant if *P* < 0.10.

We calculated that our sample size of 323 women was sufficient to detect a small effect size of 0.03 for the association between BMI and glucose, with a significance level of 5% and statistical power of at least 80%. Previously reported effect sizes were much larger (20).

Data analyses used *R (v3.5.1)* (21), graphs were produced using ggplot2 (version 3.2.1) (22) and ggpubr (version 0.0.2) (23) packages. A two-tailed *P*-value of < 0.05 was regarded significant for all analyses.

### Sensitivity Analyses

Sensitivity analyses for glucose correction (16), extreme outliers and gestational age (24–26) were carried out as a test of robustness of the results (Supplementary Materials).

## Results

The 323 women in the study had a BMI ranging from 16.6 to 41.4 kg/m^2^ and leptin levels between 1.3 and 47.1 ng/ml (Table 1). Gestational age, leptin, fasting C-peptide and the three insulin sensitivity indexes were significantly different between BMI groups, while there were no significant differences in fasting glucose between BMI groups.

### Smoking Does Not Affect Glucose, C-peptide and Insulin Sensitivity

Non-smokers were defined as those with non-smoking in self-report and cotinine levels ≤ 0.03 nmol/l (15). Based on this criterion, 125 women (38.7%) were classified as non-smokers and 198 (61.3%) women as smokers. While BMI was similar (*P* > 0.05) between the groups, leptin was higher in the non-smokers (*P* < 0.01) (Supplementary Table 1). Maternal age was also higher in the non-smokers (*P* < 0.01). Glucose and C-peptide levels as well as insulin sensitivity (IS_HOMA_) were similar in both smokers and non-smokers (*P* > 0.05).

### The Three Insulin Sensitivity Indexes Provide Similar Outcomes

Insulin sensitivity was estimated using IS_HOMA_, IS_QUICKI_ and IS_20/(FCPxFPG)_ indexes. The results were comparable for all three indexes (not shown). IS_HOMA_ has been more extensively used in the literature and, thus, will be subsequently reported.

### Glucose, C-peptide and Insulin Sensitivity Levels Change During the First Trimester of Pregnancy

Gestational age was categorized in three periods (weeks 4–6, weeks 7–9, and weeks 10–12) to test if changes of fasting glucose, fasting C-peptide and insulin sensitivity across the first trimester were linear. As the relationship was non-linear, gestational age was used as categorical variable throughout.

**Figure 1:**
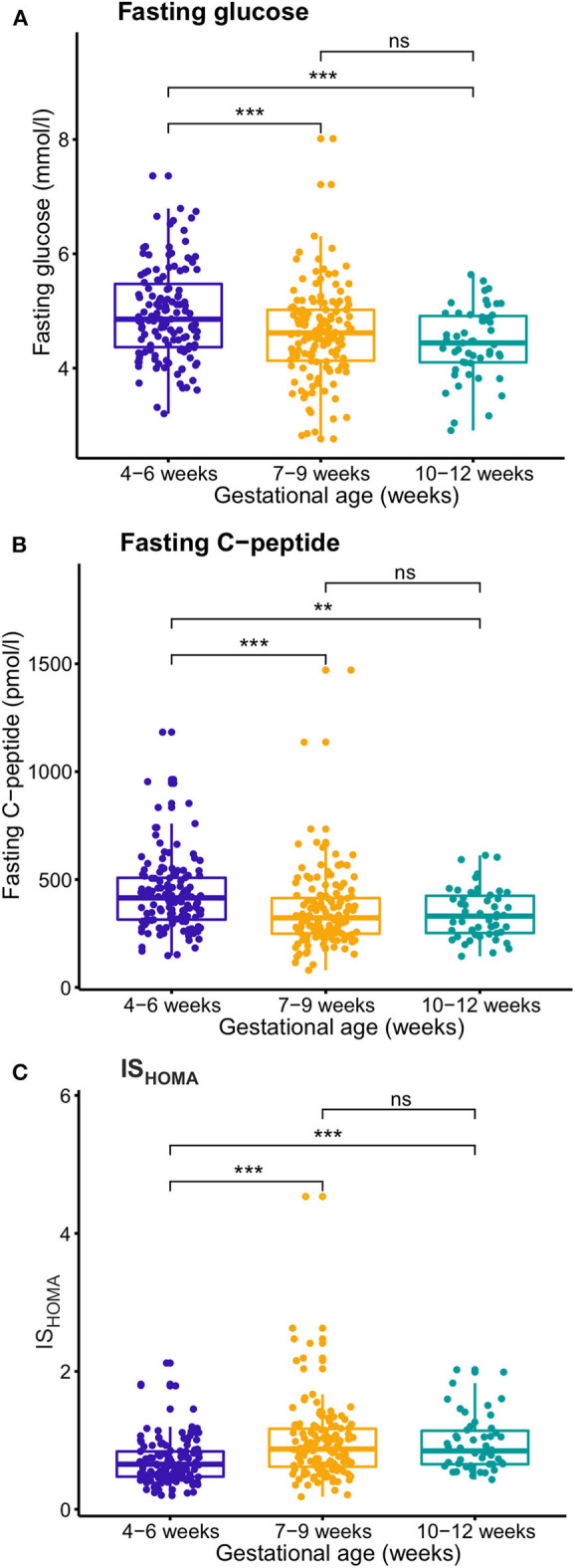
Temporal changes during the first trimester of pregnancy of fasting glucose **(A)**, fasting C-peptide **(B)**, and insulin sensitivity (IS_HOMA_) **(C)**. Gestational age was divided into three categories (weeks 4–6, 7–9, and 10–12) and glucose, C-peptide and IS_HOMA_ values were compared with ANOVA. Glucose and C-peptide decreased between weeks 4–6 and 7–9, whereas IS_HOMA_ increased. **P < 0.01, ***P < 0.001, NS, not significant.

In the unadjusted analyses, mean fasting glucose concentrations decreased between gestational weeks 4–6 and weeks 7–9, but not significantly thereafter until weeks 10–12 (Figure 1, Supplementary Table 2a). After adjusting the analyses for BMI and other confounders, results remained similar, with estimated marginal mean (EMM) fasting glucose decreasing by 6% (*P* < 0.01) between gestational weeks 4–6 (4.9 mmol/l) and weeks 7–9 (4.6 mmol/l), but remaining stable thereafter until weeks 10–12 (4.5 mmol/l) (Supplementary Table 2b).

Fasting C-peptide levels decreased from gestational weeks 4–6 to gestational weeks 7–9, but not significantly thereafter (weeks 7–9 to 10–12). After adjusting for BMI and other confounders, results remained similar, with EMM C-peptide decreasing by 18% (P < 0.001) between gestational weeks 4–6 (397.5 pmol/l) and weeks 7–9 (328.0 pmol/l), but remaining unaltered thereafter until weeks 10–12 (316.8 pmol/l). Insulin sensitivity (IS_HOMA_) increased from gestational weeks 4–6 and weeks 7–9, but not significantly thereafter (weeks 7–9 to 10–12). After adjusting for BMI and other confounders, results remained similar, with the EMM IS_HOMA_ increasing by 29% (*P* < 0.001) between gestational weeks 4–6 (0.65) and weeks 7–9 (0.84), but no further significant change thereafter (weeks 7–9 to 10–12) (0.90).

### Maternal BMI and Fat Mass Affect the Glucose-Insulin Axis in the First Trimester

BMI and measured leptin concentration in blood were used as a proxy for maternal obesity and fat mass. Serum leptin correlated with BMI (*r* = 0.510; *P* < 0.001) (Supplementary Figure 1) and was not influenced by advancing gestational age (Supplementary Table 3). Estimated marginal means of the metabolic parameters adjusted for gestational age and additional confounders are presented by BMI and leptin group in Table 2.

**Table 2:** Association between maternal BMI and leptin with fasting glucose, fasting C-peptide and insulin sensitivity (IS_HOMA_).

	**Under-/normal weight (G1)**	**Overweight (G2)**	**Obese (G3)**			
	***EEM* (95% CI)**	***EEM* (95% CI)**	***EEM* (95% CI)**	***P* G1-G2**	***P* G1-G3**	***P* G2 -G3**
Fasting glucose, mmol/l	4.7 (4.6; 4.8)	4.8 (4.6; 5.0)	4.9 (4.6; 5.2)	0.514	0.216	0.653
Fasting C- peptide, pmol/l	327.5 (311.1; 343.6)	389.6 (359.0; 428.9)	542.7 (473.2; 642.7)	**0.002**	** <0.001**	** <0.001**
IS_HOMA_	0.83 (0.78; 0.87)	0.68 (0.61; 0.75)	0.47 (0.40; 0.56)	**0.002**	** <0.001**	**0.001**
	**Leptin 1st tertile (G1)**	**Leptin 1nd tertile (G2)**	**Leptin 3rd tertile (G3)**			
	***EMM*** **(95% CI)**	***EMM*** **(95% CI)**	***EMM*** **(95% CI)**	***P*** **G1-G2**	***P*** **G1-G3**	***P*** **G2-G3**
Fasting glucose, mmol/l	4.7 (4.5; 4.8)	4.7 (4.6; 4.9)	4.8 (4.7; 4.9)	0.756	0.354	0.759
Fasting C- peptide, pmol/l	289.5 (269.4; 311.1)	363.1 (338.6; 389.3)	417.2 (388.7; 447.8)	** <0.001**	** <0.001**	**0.017**
IS_HOMA_	0.94 (0.87; 1.02)	0.74 (0.68; 0.80)	0.63 (0.58; 0.69)	** <0.001**	** <0.001**	**0.023**

*Adjusted for: gestational age (3 categories), maternal age (years), smoking and processing time (minutes). C-peptide and IS_HOMA_ log-transformed before analysis and retransformed by exponentiation. BMI, Body mass index (BMI < 25.0 kg/m^2^: under-/normal weight; BMI 25.0–29.9 kg/m^2^: overweight; BMI ≥ 30.0 kg/m^2^: obese); CI, Confidence interval; EMM, Estimated marginal means; G1, Group 1; G2, Group 2; G3, Group 3; IS_HOMA_, Homeostatic model assessment of insulin sensitivity; Leptin, (1st tertile: < 6.80 ng/ml; 2nd tertile: 6.80–12.89 ng/ml; 3rd tertile: > 12.89 ng/ml). Statistically significant results in bold*.

Glucose levels were not significantly different between BMI or leptin categories (*P* > 0.05) (Table 2). These results did not change significantly in the sensitivity analyses adjusting for 6% and 8% glucose consumption per hour (Supplementary Table 4). However, C-peptide significantly increased with increasing maternal BMI and leptin. C-peptide increased by 19% (*P* < 0.01) between the normal weight (327.5 pmol/l) and the overweight group (389.6 pmol/l) and by 39% (*P* < 0.001) between the overweight and obese group (542.7 pmol/l). In the leptin groups, C-peptide increased by 25% (*P* < 0.001) between the 1st tertile (289.5 pmol/l) and the 2nd leptin tertile (363.1 pmol/l) and by 15% (*P* < 0.05) between the 2nd and 3rd leptin tertile (417.2 pmol/l). The increase in C-peptide resulted in decreased IS_HOMA_ with higher BMI and fat mass. IS_HOMA_ decreased by 18% (*P* < 0.01) between the normal weight (0.83) and the overweight group (0.68) and by 30% (*P* < 0.01) between the overweight and the obese group (0.47). In the leptin groups, IS_HOMA_ decreased by 22% (*P* < 0.001) between the 1st tertile (0.94) and the 2nd leptin tertile (0.74) and by 14% (*P* < 0.05) between the 2nd and 3rd leptin tertile (0.63).

### No Interactions Between Maternal BMI, Leptin, Gestational Age and Smoking

To investigate whether the strength of the relationship between the metabolic parameters and BMI and leptin, respectively, differs between the gestational age groups and between smokers and non-smokers, interaction terms were added to the analyses. No significant interactions were found. Thus, while the metabolic parameters are independently associated with gestational age and maternal obesity or fat mass, no interaction was found between the variables, meaning that the relationship between the glucose-insulin axis and BMI and leptin groups is comparable during the whole first trimester of pregnancy.

### Sensitivity Analyses

Sensitivity analyses (a) applying different corrections for glucose consumption, (b) excluding outliers, and (c) testing the potential effect of misreporting gestational age provided comparable results (Supplementary Tables 4–7).

## Discussion

The present study analyzed the effect of maternal obesity and fat mass on the maternal glucose-insulin axis during the first trimester of pregnancy. The main findings were: (1) glucose and C-peptide concentrations in maternal serum decrease between week 4–6 and 7–9, paralleled by an increase in insulin sensitivity, and (2) increasing degrees of maternal obesity and fat mass were associated with higher C-peptide and lower insulin sensitivity, but not with changes in glucose levels.

To our knowledge this is the first study measuring fasting concentrations of serum glucose, C-peptide and calculating insulin sensitivity early in pregnancy (day 28 to 84) in a large sample size (323 serum samples). Furthermore, we were able to objectively distinguish smokers from non-smokers by combining self-report with cotinine levels measured in serum.

### Temporal Changes

Gestational age was statistically different between BMI groups, which could have influenced the temporal changes observed in glucose, C-peptide and IS_HOMA_. However, adjusting the analyses for BMI did not change the significance, suggesting that the differences in the glucose-insulin axis and gestational age period are independent of BMI.

Peripheral sensitivity to insulin and glucose early in pregnancy are similar to the pre-gravid period and depend on the metabolic status of the mother (27). Comparable to our study, a decrease in fasting plasma glucose during the first trimester in normal weight and overweight women was already described in a US American and a Chinese population (9, 10). The present results in a European population suggest that these changes may be independent of lifestyle and ethnicity. The decrease we found in C-peptide between week 4–6 and 7–9 is seemingly different from a longitudinal study in 34 women (28) showing an increase in insulin levels from pre-conception to week 12–14. Possible explanations could be the cross-sectional design, the much larger sample size of our study, or differences between measuring C-peptide and insulin levels. Alternatively, it may represent true physiology of an increase in fasting insulin levels between late first and early second trimester. Indeed, in a previous longitudinal time series analysis there was a trend for an increase in fasting insulin between weeks 8 and 14 (29).

The decrease in serum glucose and C-peptide concentrations we detected in early first trimester could in part be explained by a dilution effect caused by the increase in maternal plasma volume in early pregnancy as previously proposed (30). Plasma volume begins to rise at around week 6 of gestation (31), and increases by 7–10% until week 12 (32). However, if the decrease was only due to a dilution effect, then similar relative decreases in glucose and C-peptide would be expected. While the 6% decrease in glucose concentration between week 4 and 9 could be explained by the dilution effect, in the same period, C-peptide decreases by 18%. Therefore, changes in circulating fasting C-peptide levels are unlikely the sole result of plasma volume changes, and additional mechanisms must be in place. Hormones or exosomes (33) released from the placenta have been implicated in maternal adaptation to pregnancy and may contribute to the changes found here.

One of the difficulties when studying the first trimester of pregnancy is the accurate determination of gestational age. Self-reported last menstrual period is not always reliable due to irregular cycles or inability to precisely remember the date of the last menstruation (26). Ultrasound measurement of the crown-rump length (CRL), generally acknowledged as the most accurate method for the assessment of gestational age (24), is impossible or technically challenging at the earliest stages of pregnancy and we, therefore, used LMP data in our study. To accommodate these potential inaccuracies, we performed two sensitivity analyses, which confirmed robustness of the results.

The dynamic changes in fasting glucose concentrations during the first trimester have clinical relevance. They imply that screening in early pregnancy for unrecognized T2DM or GDM risk based on fasting glucose (34) would be more effective after 7–9 weeks of gestation when, based on our data, fasting glucose and fasting C-peptide concentrations have stabilized.

### Effects of Obesity and Fat Mass

We used BMI and serum leptin for the analysis of obesity and fat mass effects. The BMI was calculated from measured weight and height, avoiding self-report bias. The data were recorded prior to pregnancy termination and may in principle not accurately reflect pre-pregnancy BMI. However, body composition, objectively measured with bioelectrical impedance analysis, and BMI do not significantly change in the first trimester of pregnancy (35). Hence, the BMI of the present study can be assumed to reflect pre-pregnancy BMI.

However, BMI is a poor indicator of fat mass (36, 37) and inferior to direct measurements of visceral adipose tissue depth in explaining insulin resistance variance (38). Therefore, we measured serum leptin as a better proxy for maternal fat mass and performed all the statistical tests with both parameters. The changes observed in glucose, C-peptide and insulin sensitivity were comparable between BMI and leptin categories, which add confidence that the BMI-associated differences are the result of fat mass differences. Although we cannot disregard some placental contribution to maternal leptin levels in the first trimester, we argue that most of the leptin measured here was secreted by maternal adipose tissue, hence reflecting maternal fat mass, because (1) maternal serum leptin correlated with maternal BMI, and (2) serum leptin did not increase with increasing gestational age, not even after adjusting for BMI.

The higher maternal fat mass, measured as leptin, was associated with higher C-peptide and lower insulin sensitivity. Higher insulin levels and insulin resistance in obese as compared to normal weight has also been reported before in a small cohort measured at one time point (mean gestational age week 9) (39).

Smoking alters metabolism in a number of ways, including changes in plasma lipids and increased insulin resistance (13, 14). Interestingly, in our study smoking did not significantly influence the glucose-insulin axis. However, leptin levels were lower in the smokers. This has been described before outside of pregnancy (40) and several mechanisms have been suggested to explain this phenomenon (36, 37, 41, 42).

### Strengths and Limitations

A specific strength is the inclusion of the very early weeks of gestation, because of the predominant histiotrophic route of nourishing the embryo/feto-placental unit. This may require higher maternal concentrations of key metabolites as compared to the later stages in the first trimester, when the maternal circulation supplies its nutrients directly to the feto-placental unit.

The cohort comprised women, who intentionally had their pregnancies terminated for psychosocial reasons. Smoking women are overrepresented (61%) compared to the average prevalence of female smokers (22%) in Austria (43). Both aspects may limit representativeness of the results. As we do not have socio-economic information on the women, we could not adjust for this in the analyses.

IS_HOMA_ is the most widely used index for insulin sensitivity and IS_HOMA_ correlates with glucose clamp results, the gold standard for measuring insulin sensitivity (44), but there is evidence that it might not be the most suitable in all populations and ethnicities. For example it is of limited use in subjects with low BMI, reduced β-cell function or high fasting plasma glucose (45). We lack information regarding the ethnicity of our study population. Thus, to avoid bias by limiting the calculation of insulin sensitivity to IS_HOMA_, we used three different indexes IS_HOMA_, IS_QUICKI_ and IS_20/(FCPxFPG)_ (17, 18) as a proxy for maternal insulin sensitivity, and all three provided essentially similar results.

A limitation is the non-academic setting, which precluded spinning the blood immediately after collection. Hence, we had to introduce a correction factor for glucose consumption by red blood cells. However, sensitivity analyses using different correction values did not significantly change the results. For diabetes diagnosis, collection of plasma instead of serum is preferred, since it might improve the diagnosis in those patients with glucose levels close to the cut-off values (46). In our study, we excluded patients with known comorbidities such as pre-existing diabetes. Therefore, the use of serum or plasma for glucose quantification should not have affected the results. Another limitation is the cross-sectional design, which did not allow testing changes of metabolic parameters within different subgroups over time. Therefore, we could not assess whether metabolic heterogeneity of obesity may have influenced the results, which are limited to the group level.

Finally, fetal sex should have been considered, because carrying a male fetus is associated with higher fasting glucose in the mother (47) and an increased risk of developing GDM (48). However, we did not have placental tissue available for all subjects for genotyping and thus could not test sex-effects in our study.

## Conclusions

The first trimester of pregnancy is a dynamic period that requires metabolic adaptations. We have shown differences in fasting glucose, fasting C-peptide and IS_HOMA_ even within the narrow time window of 8 weeks, i.e., week 4–12, covered in the present study. Furthermore, as we demonstrate for the first time, maternal obesity and higher fat mass are associated with higher fasting C-peptide and lower insulin sensitivity throughout the first trimester of human pregnancy. This suggests impairment in the glucose-insulin axis already very early in the first trimester in obese women. As a speculation, early dysregulation in the glucose-insulin axis may link maternal obesity/fat mass with altered placental development (49) and contribute to adverse pregnancy outcomes in obese mothers. The variation in the glucose-insulin axis in the first trimester both with time and maternal fat mass may explain the poor predictive power of early fasting blood glucose for later GDM. Based on the present study, GDM risk is best assessed in the last weeks of the first trimester of pregnancy, when the glucose-insulin axis has stabilized. The dynamic changes shown here call for future studies encompassing a wider range of metabolites and hormones to improve the understanding of the physiologic mechanisms underlying early metabolic and endocrine adaptive responses of the mother to a beginning pregnancy.

## Data Availability Statement

The raw data supporting the conclusions of this article will be made available by the authors upon reasonable request.

## Ethics Statement

The studies involving human participants were reviewed and approved by Ethical Committee of the Medical University of Graz. The patients/participants provided their written informed consent to participate in this study.

## Author Contributions

JB-M: investigation, data curation, formal analysis, visualization, and writing original draft. AD: data curation, formal analysis, visualization, and writing original draft. DH, CP, MB, and TN: investigation, writing-review and editing. AG: resources and writing-review and editing. MP: formal analysis and writing-review and editing. GD: conceptualization, funding acquisition, supervision, and writing-review and editing. All authors contributed to the article and approved the submitted version.

## Conflict of Interest

The authors declare that the research was conducted in the absence of any commercial or financial relationships that could be construed as a potential conflict of interest.
