# Supplementary material for: Maternal Obesity Affects the Glucose-Insulin Axis During the First Trimester of Human Pregnancy
Source: Front Endocrinol (Lausanne). 2020 Oct 9;11:566673. doi: 10.3389/fendo.2020.566673 (PMC7586307; doi:10.3389/fendo.2020.566673)
Supplement: Supplementary file 1 [file Data_Sheet_1.pdf]

**Supplementary Table 1.** Characteristics of smokers and non-smokers participants. Maternal age and leptin (ng/ml) were significantly different between smokers and non-smokers.

| Maternal Characteristics | N | Smokers (N = 198) | Non-smokers (N = 125) | *P* |
|---|---|---|---|---|
| Age, *years* (mean ± SD) | 322 | **28.2 ± 6.6** | **31.5 ± 7.1** | **< 0.001** |
| Gestational age [N (%)] | 323 | | | 0.346 |
| 4 - 6 weeks | | 76 (38.4) | 58 (46.4) | |
| 7 - 9 weeks | | 90 (45.4) | 48 (38.4) | |
| 10 - 12 weeks | | 32 (16.2) | 19 (15.2) | |
| BMI, *kg/m²* [median (IQR)] | | 22.6 (20.2 - 25.3) | 22.8 (20.8 - 25.7) | 0.383 |
| Leptin, *ng/ml* (median (IQR)) | 321 | **8.7 (4.1 - 13.5)** | **11.9 (7.2 - 17.4)** | **< 0.001** |
| Fasting glucose, *mmol/l* (mean ± SD) | 323 | 4.7 ± 0.8 | 4.6 ± 0.9 | 0.662 |
| Fasting C-peptide, *pmol/l* [median (IQR)] | 322 | 341.3 (264.2 –448.8) | 371.9 (281.3 –472.1) | 0.108 |
| $IS_{HOMA}$ [median (IQR)] | 322 | 0.79 (0.56 - 1.05) | 0.73 (0.53 - 1.00) | 0.131 |
| $IS_{20/(FCPxFPG)}$ [median (IQR)] | 322 | 12.60 (9.0 - 16.80) | 11.60 (8.50 - 16.00) | 0.131 |
| $IS_{QUICKI}$ (mean ± SD) | 322 | 0.23 ± 0.01 | 0.22 ± 0.01 | 0.144 |

BMI, leptin, C-peptide, $IS_{HOMA}$ and $IS_{20/(FCPxFPG)}$ log-transformed before t-test. BMI: Body mass index; SD: standard deviation; IQR: interquartile range. Statistically significant results in bold.

**Supplementary Table 2a.** Fasting glucose, fasting C-peptide and insulin sensitivity ($IS_{HOMA}$) by gestational age. Results graphically displayed in Figure 1.

| | **GA week 4-6 (T1)** *Mean (95% CI)* | **GA week 7-9 (T2)** *Mean (95% CI)* | **GA week 10-12 (T3)** *Mean (95% CI)* | *P T1-T2* | *P T1-T3* | *P T2 -T3* |
|---|---|---|---|---|---|---|
| Fasting glucose, *mmol/l* | 5.0 (4.8; 5.1) | 4.6 (4.5; 4.7) | 4.4 (4.2; 4.7) | **< 0.001** | **< 0.001** | 0.371 |
| Fasting C- peptide, *pmol/l* | 402.0 (376.0; 429.9) | 323.0 (302.4; 345.1) | 323.3 (289.8; 360.8) | **< 0.001** | **0.003** | > 0.999 |
| $IS_{HOMA}$ | 0.64 (0.59; 0.69) | 0.85 (0.79; 0.92) | 0.88 (0.78; 1.00) | **< 0.001** | **< 0.001** | 0.904 |

Fasting glucose, fasting C-peptide and $IS_{HOMA}$ compared by analysis of variance (ANOVA) and Tukey post hoc test. C-peptide and $IS_{HOMA}$ log-transformed before analysis and retransformed by exponentiation. CI: confidence interval, GA: gestational age; $IS_{HOMA}$: Homeostatic model assessment of insulin sensitivity; T1: Time 1; T2: Time 2; T3: Time 3. Statistically significant results in bold.

**Supplementary Table 2b.** Fasting glucose, fasting C-peptide and insulin sensitivity ($IS_{HOMA}$) by gestational age, adjusted for confounders.

| | **GA week 4-6 (T1)** *EMM (95% CI)* | **GA week 7-9 (T2)** *EMM (95% CI)* | **GA week 10-12 (T3)** *EMM (95% CI)* | *P T1-T2* | *P T1-T3* | *P T2 -T3* |
|---|---|---|---|---|---|---|
| Fasting glucose, *mmol/l* | 4.9 (4.8; 5.1) | 4.6 (4.4; 4.8) | 4.5 (4.2; 4.7) | **0.003** | **< 0.001** | 0.351 |
| Fasting C- peptide, *pmol/l* | 397.5 (373.2; 423.3) | 328.0 (308.2; 349.0) | 316.8 (285.5; 351.4) | **< 0.001** | **< 0.001** | 0.838 |
| $IS_{HOMA}$ | 0.65 (0.60; 0.70) | 0.84 (0.78; 0.90) | 0.90 (0.79; 1.01) | **< 0.001** | **< 0.001** | 0.602 |

Fasting glucose, fasting C-peptide and $IS_{HOMA}$ compared by analysis of covariance (ANCOVA) and Tukey post hoc test. Adjusted for: BMI (3 categories), maternal age (years), smoking and processing time (minutes). C-peptide and $IS_{HOMA}$ log-transformed before analysis and retransformed by exponentiation. Statistically significant results in bold. BMI: Body mass index; CI: confidence interval; EMM: Estimated marginal means; GA: gestational age; $IS_{HOMA}$: Homeostatic model assessment of insulin sensitivity; T1: Time 1; T2: Time 2; T3: Time 3. Statistically significant results in bold.

**Supplementary Table 3**. BMI and serum leptin are not associated with gestational age in multivariate regression analysis.

| | | **BMI (ln)** | | **Leptin (ln)** | |
|---|---|---|---|---|---|
| | | *Estimate (95% CI)* | *P* | *Estimate (95% CI)* | *P* |
| Gestational age (days) | Model 1 | 0.000 (-0.001; 0.001) | 0.789 | -0.003 (-0.008; 0.003) | 0.361 |
| | Model 2 | 0.000 (-0.001; 0.001) | 0.421 | -0.003 (-0.008; 0.002) | 0.233 |

Model 1: Adjusted for smoking and maternal age (years); Model 2: Additionally adjusted for BMI (ln) / leptin (ln). BMI: Body mass index; CI: Confidence interval.

**Supplementary Figure 1**. Spearman’s correlation between BMI and Leptin.

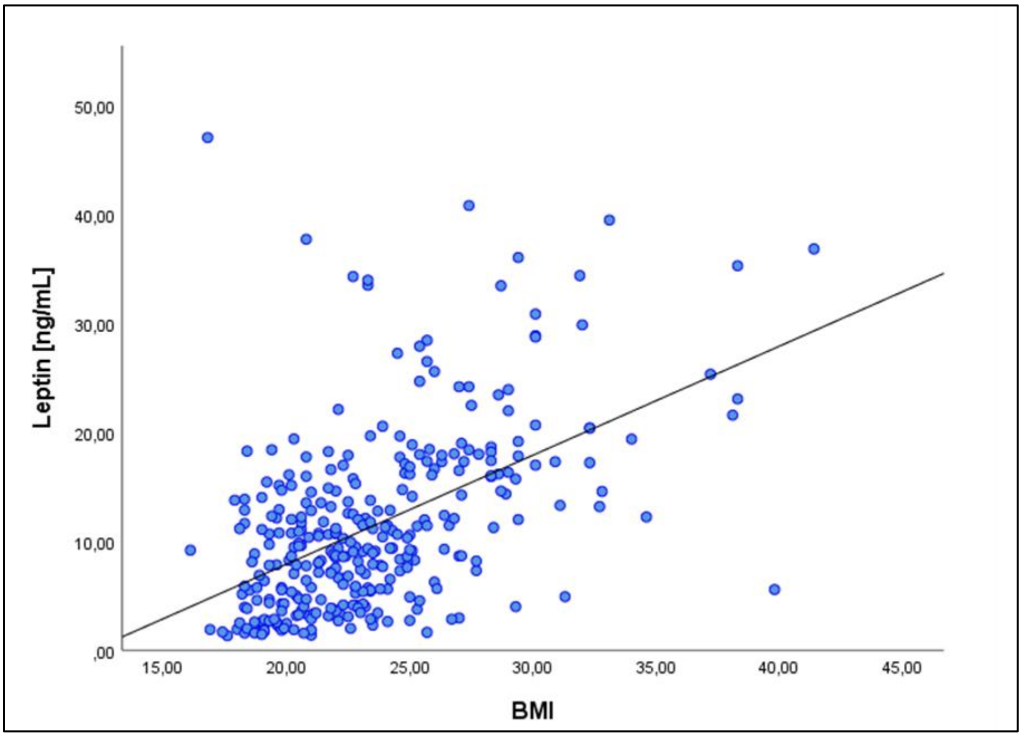


r= 0.510; $P < 0.001$

## SENSITIVITY ANALYSES

### Methods

Several sensitivity analyses were carried out as a test of robustness of the results.

a) Sensitivity analysis for glucose correction

For the main analyses, fasting glucose levels were adjusted for a 7% decrease per hour. To test the influence of this adjustment, sensitivity analyses were done with alternatively a 6% and 8% correction per hour.

b) Sensitivity analysis for extreme outliers

To test whether any outliers of metabolic parameters influenced the results, extreme outliers detected by two researchers by visual inspection (C-peptide > 1.300 nmol/l and $IS_{HOMA}$ > 4) were excluded for two additional sensitivity analyses.

c) Sensitivity analysis for gestational age

Generally, it is agreed that gestational age can be determined most accurately by an early ultrasound measurement of the embryo or fetus in the first trimester of pregnancy (Committee opinion 700, 2017). However, as our study includes very early pregnancies (< 6 weeks of gestation) where the embryo is often not yet visible, LMP was chosen as the preferred method to determine gestational age. Self-reported LMP, however, can be unreliable due to potential misreporting and also because obesity may affect ovulation (Hall, Carr-Hill, Fraser, Campbell, & Samphier, 1985). To assess the robustness of the LMP-based gestational age, a sensitivity analysis was conducted by repeating all analyses with gestational age based on ultrasonic crown-rump length measurement (CRL) (Loughna, Chitty, Evans, & Chudleigh, 2009). As a second test, random noise between -3 and +3 days was added to the LMP-based gestational age and analyses were repeated. Initial analyses used gestational age based on LMP.

## Results

a) Sensitivity analysis for glucose correction

Using either 6% or 8% correction per hour for fasting glucose produced comparable results and did not change significance (**Supplementary Table 4**).

b) Sensitivity analysis for extreme outliers

The effect of BMI and fat mass (**Table 2**) was comparable and did not change significance in fasting glucose, fasting C-peptide and $IS_{HOMA}$ after excluding extreme outliers (**Supplementary Table 5**). The influence of BMI and fat mass on fasting glucose, fasting C-peptide and $IS_{HOMA}$ across gestation (**Figure 1, Supplementary Table 2**) was comparable after exclusion of extreme outliers (**Supplementary Table 6**) and did not result in changes in significance.

c) Sensitivity analysis for gestational age

Addition of random noise to last menstrual period (LMP) based gestation age (**Supplementary Table 7 A**) and re-calculating gestational age based on crown-rump length (CRL) measurements (**Supplementary Table 7 B**) produced comparable results and did not change significance.

## Bibliography


1. Committee opinion 700. Methods for estimating the Due Date. Obstet Gynecol. 2017;129(5):959–60
2. Hall MH, Carr-Hill RA, Fraser C, Campbell D, Samphier ML. The extent and antecedents of uncertain gestation. Br J Obstet Gynaecol. 1985;92(5):445–51.
3. Loughna P, Chitty L, Evans T, Chudleigh T. Fetal size and dating: Charts recommended for clinical obstetric practice. Ultrasound. 2009;17(3):160–6.

**Supplementary Table 4.** Sensitivity analysis for the analysis of covariance applying 6% (A) and 8% (B) per hour (hr) correction for the glucose consumption rate (see Table 2 for original analysis).

**A)**

| | **Under-/normal weight (G1)** *EEM (95% CI)* | **Overweight (G2)** *EEM (95% CI)* | **Obese (G3)** *EEM (95% CI)* | *P G1-G2* | *P G1-G3* | *P G2 -G3* |
|---|---|---|---|---|---|---|
| Fasting glucose, *mmol/l* | 4.5 (4.4; 4.6) | 4.6 (4.4; 4.8) | 4.8 (4.5; 5.0) | 0.528 | 0.215 | 0.644 |
| | **Leptin 1st tertile (G1)** *EEM (95% CI)* | **Leptin 2nd tertile (G2)** *EMM (95% CI)* | **Leptin 3rd tertile (G3)** *EMM (95% CI)* | *P G1-G2* | *P G1-G3* | *P G2 -G3* |
| Fasting glucose, *mmol/l* | 4.5 (4.3; 4.6) | 4.5 (4.4; 4.7) | 4.6 (4.5; 4.8) | 0.779 | 0.361 | 0.743 |

**B)**

| | **Under-/normal weight (G1)** *EEM (95% CI)* | **Overweight (G2)** *EEM (95% CI)* | **Obese (G3)** *EEM (95% CI)* | *P G1-G2* | *P G1-G3* | *P G2 -G3* |
|---|---|---|---|---|---|---|
| Fasting glucose, *mmol/l* | 4.9 (4.8; 5.0) | 5.0 (4.8; 5.2) | 5.1 (4.8; 5.5) | 0.502 | 0.218 | 0.663 |
| | **Leptin 1st tertile (G1)** *EMM (95% CI)* | **Leptin 2nd tertile (G2)** *EMM (95% CI)* | **Leptin 3rd tertile (G3)** *EMM (95% CI)* | *P G1-G2* | *P G1-G3* | *P G2 -G3* |
| Fasting glucose, *mmol/l* | 4.8 (4.7; 5.0) | 4.9 (4.8; 5.1) | 5.0 (4.8; 5.1) | 0.733 | 0.350 | 0.777 |

Adjusted for: gestational age (3 categories), maternal age (years), smoking and processing time (minutes). Fasting C-peptide and $IS_{HOMA}$ log-transformed before analysis and retransformed by exponentiation. BMI: Body mass index (BMI < 25.0 kg/$m^2$: under-/normal weight; BMI 25.0 – 29.9: overweight; BMI ≥ 30.0 kg/$m^2$: obese); CI: Confidence interval; EMM: Estimated marginal means; G1: Group 1; G2: Group 2; G3: Group 3; $IS_{HOMA}$: Homeostatic model assessment of insulin sensitivity. Leptin: (1st tertile: < 6.80 ng/ml; 2nd tertile: 6.80-12.89 ng/ml; 3rd tertile: > 12.89 ng/ml). Statistically significant results in bold.

**Supplementary Table 5.** Sensitivity analysis for the analysis of covariance excluding extreme outliers (see Table 2 for original analysis).

| | **Under-/normal weight (G1)** *EEM (95% CI)* | **Overweight (G2)** *EEM (95% CI)* | **Obese (G3)** *EEM (95% CI)* | *P G1-G2* | *P G1-G3* | *P G2 -G3* |
|---|---|---|---|---|---|---|
| Fasting glucose, *mmol/l* | 4.7 (4.6; 4.8) | 4.8 (4.6; 5.0) | 5.0 (4.7; 5.3) | 0.555 | 0.159 | 0.527 |
| Fasting C- peptide, *pmol/l* | 325.7 (311.2; 340.9) | 389.6 (359.0; 422.8) | 550.7 (478.1; 634.4) | **0.001** | **< 0.001** | **< 0.001** |
| $IS_{HOMA}$ | 0.83 (0.79; 0.88) | 0.68 (0.62; 0.75) | 0.46 (0.39; 0.55) | **< 0.001** | **< 0.001** | **< 0.001** |
| | **Leptin 1st tertile (G1)** *EEM (95% CI)* | **Leptin 2nd tertile (G2)** *EMM (95% CI)* | **Leptin 3rd tertile (G3)** *EMM (95% CI)* | *P G1-G2* | *P G1-G3* | *P G2 -G3* |
| Fasting glucose, *mmol/l* | 4.7 (4.5; 4.8) | 4.7 (4.6; 4.9) | 4.8 (4.7; 5.0) | 0.854 | 0.444 | 0.745 |
| Fasting C- peptide, *pmol/l* | 292.5 (272.9; 313.6) | 362.7 (339.4; 387.7) | 408.6 (381.7; 437.5) | **< 0.001** | **< 0.001** | **0.037** |
| $IS_{HOMA}$ | 0.93 (0.86; 1.01) | 0.74 (0.68; 0.80) | 0.65 (0.60; 0.70) | **< 0.001** | **< 0.001** | **0.047** |

Adjusted for: gestational age (3 categories), maternal age (years), smoking and processing time (minutes). C-peptide and $IS_{HOMA}$ log-transformed before analysis and retransformed by exponentiation. BMI: Body mass index (BMI < 25.0 kg/m$^2$: under-/normal weight; BMI 25.0 – 29.9: overweight; BMI ≥ 30.0 kg/m$^2$: obese); CI: Confidence interval; EMM: Estimated marginal means; G1: Group 1; G2: Group 2; G3: Group 3; $IS_{HOMA}$: Homeostatic model assessment of insulin sensitivity. Leptin: (1st tertile: < 6.80 ng/ml; 2nd tertile: 6.80-12.89 ng/ml; 3rd tertile: > 12.89 ng/ml). Statistically significant results in bold.

**Supplementary Table 6.** Sensitivity analysis excluding extreme outliers for fasting glucose, fasting C-peptide and insulin sensitivity ($IS_{HOMA}$) (see Supplementary table 2b for original analysis).

| | **Week 4-6 (T1)** *Mean (95% CI)* | **Week 7-9 (T2)** *Mean (95% CI)* | **Week 10-12 (T3)** *Mean (95% CI)* | *P T1-T2* | *P T1-T3* | *P T2 -T3* |
|---|---|---|---|---|---|---|
| Fasting glucose, *mmol/l* | 4.9 (4.8; 5.1) | 4.6 (4.5; 4.8) | 4.5 (4.3; 4.7) | **0.004** | < **0.001** | 0.312 |
| Fasting C- peptide, *pmol/l* | 395.5 (372.7; 419.7) | 327.4 (308.7; 347.1) | 315.8 (286.6; 348.1) | < **0.001** | **<0.001** | 0.808 |
| $IS_{HOMA}$ | 0.65 (0.61; 0.70) | 0.84 (0.78; 0.90) | 0.90 (0.80; 1.01) | < **0.001** | < **0.001** | 0.538 |

Fasting glucose, fasting C-peptide and $IS_{HOMA}$ compared by analysis of covariance (ANCOVA) and Tukey post hoc test. Adjusted for: BMI (3 categories), maternal age (years), smoking and processing time (minutes). C-peptide and $IS_{HOMA}$ log-transformed before analysis and retransformed by exponentiation. Statistically significant results in bold. BMI: Body mass index; CI: confidence interval; EMM: Estimated marginal means; $IS_{HOMA}$: Homeostatic model assessment of insulin sensitivity; T1: Time 1; T2: Time 2; T3: Time 3. Statistically significant results in bold.

**Supplementary Table 7.** Sensitivity analysis for the analysis of covariance with gestational age calculated from the ultrasound-measured CRL (A) and with the addition of random noise (± 3 days interval) to the gestational age based on self-reported LMP (B). (See Table 2 for original analysis).

**A)**

| | **Under-/normal weight (G1)** *EEM (95% CI)* | **Overweight (G2)** *EEM (95% CI)* | **Obese (G3)** *EEM (95% CI)* | *P G1-G2* | *P G1-G3* | *P G2 -G3* |
|---|---|---|---|---|---|---|
| Fasting glucose, *mmol/l* | 4.7 (4.6; 4.8) | 4.8 (4.6; 5.0) | 5.0 (4.7; 5.3) | 0.524 | 0.176 | 0.592 |
| Fasting C- peptide, *pmol/l* | 327.2 (311.7; 343.4) | 388.3 (355.5; 424.1) | 547.9 (472.7; 635.1) | **0.003** | **< 0.001** | **< 0.001** |
| $IS_{HOMA}$ | 0.83 (0.78; 0.88) | 0.68 (0.62; 0.76) | 0.47 (0.39; 0.55) | **0.003** | **< 0.001** | **< 0.001** |
| | **Leptin 1st tertile (G1)** *EEM (95% CI)* | **Leptin 2nd tertile (G2)** *EMM (95% CI)* | **Leptin 3rd tertile (G3)** *EMM (95% CI)* | *P G1-G2* | *P G1-G3* | *P G2 -G3* |
| Fasting glucose, *mmol/l* | 4.7 (4.5; 4.8) | 4.7 (4.6; 4.9) | 4.8 (4.7; 4.9) | 0.708 | 0.318 | 0.764 |
| Fasting C- peptide, *pmol/l* | 288.6 (268.6; 310.2) | 363.9 (339.3; 390.2) | 416.7 (388.1; 447.4) | **< 0.001** | **< 0.001** | **0.021** |
| $IS_{HOMA}$ | 0.95 (0.87; 1.03) | 0.74 (0.68; 0.80) | 0.63 (0.58; 0.55) | **<0.001** | **< 0.001** | **0.029** |

Adjusted for: gestational age (3 categories), maternal age (years), smoking and processing time (minutes). C-peptide and $IS_{HOMA}$ log-transformed before analysis and retransformed by exponentiation. BMI: Body mass index (BMI < 25.0 $kg/m^2$: under-/normal weight; BMI 25.0 – 29.9: overweight; BMI ≥ 30.0 $kg/m^2$: obese); CRL: Crown-rump length; CI: Confidence interval; EMM: Estimated marginal means; G1: Group 1; G2: Group 2; G3: Group 3; $IS_{HOMA}$: Homeostatic model assessment of insulin sensitivity; Leptin: (1st tertile: < 6.80 ng/ml; 2nd tertile: 6.80-12.89 ng/ml; 3rd tertile: > 12.89 ng/ml); LMP: Last menstrual period. Statistically significant results in bold.

**B)**

| | **Under-/normal weight (G1)** *EEM (95% CI)* | **Overweight (G2)** *EEM (95% CI)* | **Obese (G3)** *EEM (95% CI)* | *P G1-G2* | *P G1-G3* | *P G2 -G3* |
|---|---|---|---|---|---|---|
| Fasting glucose, *mmol/l* | 4.7 (4.6; 4.8) | 4.8 (4.6; 4.9) | 5.0 (4.7; 5.3) | 0.779 | 0.209 | 0.493 |
| Fasting C- peptide, *pmol/l* | 326.8 (311.4; 343.0) | 394.8 (361.3; 431.4) | 545.9 (470.9; 632.9) | **< 0.001** | **< 0.001** | **< 0.001** |
| $IS_{HOMA}$ | 0.83 (0.78; 0.88) | 0.68 (0.61; 0.75) | 0.47 (0.40; 0.56) | **0.002** | **< 0.001** | **0.001** |
| | **Leptin 1st tertile (G1)** *EEM (95% CI)* | **Leptin 2nd tertile (G2)** *EMM (95% CI)* | **Leptin 3rd tertile (G3)** *EMM (95% CI)* | *P G1-G2* | *P G1-G3* | *P G2 -G3* |
| Fasting glucose, *mmol/l* | 4.7 (4.5; 4.8) | 4.7 (4.6; 4.9) | 4.8 (4.7; 4.9) | 0.805 | 0.356 | 0.713 |
| Fasting C- peptide, *pmol/l* | 289.8 (269.6; 311.6) | 364.4 (339.3; 391.2) | 417.4 (388.7; 448.3) | **< 0.001** | **< 0.001** | **0.022** |
| $IS_{HOMA}$ | 0.94 (0.87; 1.03) | 0.74 (0.68; 0.80) | 0.63 (0.58; 0.69) | **< 0.001** | **< 0.001** | **0.029** |

Adjusted for: gestational age (3 categories), maternal age (years), smoking and processing time (minutes). C-peptide and $IS_{HOMA}$ log-transformed before analysis and retransformed by exponentiation. BMI: Body mass index (BMI < 25.0 kg/m$^2$: under-/normal weight; BMI 25.0 – 29.9: overweight; BMI ≥ 30.0 kg/m$^2$: obese); CRL: Crown-rump length; CI: Confidence interval; EMM: Estimated marginal means; G1: Group 1; G2: Group 2; G3: Group 3; $IS_{HOMA}$: Homeostatic model assessment of insulin sensitivity; Leptin: (1st tertile: < 6.80 ng/ml; 2nd tertile: 6.80-12.89 ng/ml; 3rd tertile: > 12.89 ng/ml); LMP: Last menstrual period. Statistically significant results in bold.
